# Brazilian Propolis Suppresses Angiogenesis by Inducing Apoptosis in Tube-Forming Endothelial Cells through Inactivation of Survival Signal ERK1/2

**DOI:** 10.1093/ecam/nep024

**Published:** 2010-10-31

**Authors:** Kazuhiro Kunimasa, Mok-Ryeon Ahn, Tomomi Kobayashi, Ryoji Eguchi, Shigenori Kumazawa, Yoshihiro Fujimori, Takashi Nakano, Tsutomu Nakayama, Kazuhiko Kaji, Toshiro Ohta

**Affiliations:** ^1^Graduate School of Nutritional and Environmental Sciences and Global COE Program, University of Shizuoka, Shizuoka 422-8526, Japan; ^2^Department of Food and Nutrition, Dong-A University, 840 Hadan-2 dong, Saha-gu, Busan 604-714, Republic of Korea; ^3^Department of Thoracic Oncology, Hyogo College of Medicine, Japan; ^4^Cancer Center, Hyogo College of Medicine, Hyogo 663-8501, Japan

## Abstract

We recently reported that propolis suppresses tumor-induced angiogenesis through tube formation inhibition and apoptosis induction in endothelial cells. However, molecular mechanisms underlying such angiogenesis suppression by propolis have not been fully elucidated. The aim of this study was to investigate the effects of ethanol extract of Brazilian propolis (EEBP) on two major survival signals, extracellular signal-regulated kinase 1/2 (ERK1/2) and Akt, and to elucidate whether changes in these signals were actually involved in antiangiogenic effects of the propolis. Detection by western blotting revealed that EEBP suppressed phosphorylation of ERK1/2, but not that of Akt. Pharmacological inhibition by U0126 demonstrated that ERK1/2 inactivation alone was enough to inhibit tube formation and induce apoptosis. It was also shown that EEBP and U0126 similarly induced activation of caspase-3 and cleavage of poly ADP-ribose polymerase (PARP) and lamin A/C, all of which are molecular markers of apoptosis. These results indicate that inhibition of survival signal ERK1/2, and subsequent induction of apoptosis, is a critical mechanism of angiogenesis suppression by EEBP.

## 1. Introduction

Propolis is a resinous substance collected by honeybees from buds and exudates of certain trees and plants, and stored inside their hives. It has been used in folk medicine from ancient times to treat various ailments [1, 2]. It has been revealed that propolis possesses various biological activities such as antibacterial [3, 4], antifungal [3, 4], antiviral [3, 5], anti-inflammatory [6] and anticancer [7–10] properties. We previously reported that ethanol extract of Brazilian propolis (EEBP) suppresses tumor-induced angiogenesis *invivo* and tube formation of endothelial cells *invitro* [11]. We also demonstrated recently that propolis suppresses angiogenesis through induction of apoptosis in endothelial cells, but molecular mechanisms underlying induction of endothelial cell apoptosis by propolis have not been fully elucidated [12].

Angiogenesis is defined as the process in which a network of new blood vessels emerges from pre-existing vessels. Angiogenesis has been shown to be essential for tumor growth and metastasis, which are two major factors that hinder cancer therapy [13]. We and others have shown that food factors, such as epigallocatechin gallate (EGCg), indole-3-carbinol, resveratrol and quercetin, possessed antiangiogenic properties [14–17]. Such antiangiogenic food factors could be used to effectively prevent small cancers from progressing.

Investigation of the effects of many angiogenesis inhibitors has revealed that one of the major antiangiogenic mechanisms of these drugs is induction of apoptosis in endothelial cells [18]. Apoptosis is a genetically programmed form of cell death. Angiogenic stimuli such as vascular endothelial growth factor (VEGF) and basic fibroblast growth factor (bFGF) are known to activate extracellular signal-regulated kinase 1/2 (ERK1/2) and Akt, which transduce survival signals in endothelial cells and simultaneously prevent apoptosis by inactivating proapoptotic proteins [19–21]. On the other hand, apoptotic stimuli are known to activate a caspase cascade that ultimately leads to the oligonucleosomal fragmentation of DNA and the cleavage of proteins such as poly ADP-ribose polymerase (PARP) and lamin A/C [22].

In this study, we investigated the effects of EEBP on endothelial cell apoptosis. We also analyzed changes in survival signals using western blotting. We further investigated the role of ERK1/2 inactivation by the propolis using U0126, a specific pharmacological inhibitor of ERK1/2 activation.

## 2. Materials and Methods

### 2.1. Materials

Medium MCDB-104 was purchased from Nihon Pharmaceutical (Tokyo, Japan), fetal bovine serum (FBS) from Moregate (Brisbane, Australia), Atelocollagen Bovine Dermis (type I collagen) from Koken (Tokyo, Japan), human bFGF (Recombinant) from Austral Biologicals (San Ramon, CA) and U0126 from Calbiochem (La Jolla, MO). Unless otherwise stated, all chemicals were purchased from Sigma (St Louis, MO). All antibodies used in this experiment were from Cell Signaling Technology (Beverly, MA). EGCg, which was used as a positive control food factor to inhibit angiogenesis, was a kind gift from Dr Yukihiko Hara at Tokyo Food Techno Co., Ltd. (Tokyo, Japan) and dissolved in sodium phosphate buffer (pH 6.0).

### 2.2. Preparation of Propolis Extract

Brazilian propolis was collected in Minas Gerais State, where *Baccharis dracunculifolia* DC. is the main botanical source of the propolis. Propolis sample was extracted with ethanol (30 ml of ethanol per gram of propolis) at room temperature for 24 h as previously reported [23]. The ethanol suspension was separated by centrifugation at 470 × g for 15 min at 25°C, and the supernatant was concentrated in a rotary evaporator under reduced pressure at 40°C until reaching a constant weight, and then redissolved in ethanol. The preparation obtained was named ethanol extract of Brazilian propolis (EEBP), and the color of EEBP was brown. EEBP was stored under a dry condition at 4°C until analyzed.

### 2.3. Tube Formation Assay

Human umbilical vein endothelial cells (HUVECs) were grown in MCDB-104 supplemented with 10% FBS, 10 ng ml^−1^ EGF, 100 *μ*g ml^−1^ heparin and 100 ng ml^−1^ endothelial cell growth factor as previously reported [16]. The cells were seeded on plates coated with 0.1% gelatin and allowed to grow to sub-confluence at 37°C.

Capillary tube-like structures formed by HUVECs in collagen gel were prepared as previously described with slight modifications [11]. Briefly, HUVECs (6.0 × 10^4^ cells cm^−2^) were seeded in between two layers of collagen gel (0.21% collagen) and incubated in MCDB-104 supplemented with 0.5% FBS, 10 ng ml^−1^ bFGF, 8 nM phorbol 12-myristate 13-acetate (PMA) and 25 *μ*g ml^−1^ ascorbic acid. They were treated with various concentrations of EEBP (0, 6.25, 12.5 and 25 *μ*g ml^−1^), EGCg (50 *μ*g ml^−1^) or U0126 (5 *μ*M) for up to 36 h. The tube formation was quantified by determining the pixel number of tubes in each image using the NIH Image program (developed at the US National Institutes of Health and available on the Internet at http://rsb.info.nih.gov/nih-image/).

For western blot analysis, HUVECs (2.4 × 10^5^ cells per 48-well) were suspended three-dimensionally in collagen gel, instead of being sandwiched between two layers of collagen gel, for 12 and 24 h.

### 2.4. Apoptosis

Observation and quantification of apoptosis were carried out as previously described [21]. Briefly, after experimental treatment, the cells were fixed with 1% glutaraldehyde overnight at 4°C and stained with 500 ng/ml^−1^ of 4′,6-diamidino-2-phenylindole (DAPI) overnight at room temperature. Cells exhibiting chromatin condensation and/or cell nuclear fragmentation were counted as apoptotic cells. A total of more than 700 cells from six fields were counted for each data.

### 2.5. Western Blot Analysis

Western blotting was carried out as previously described [24]. Briefly, the cells in collagen gel after experimental treatment were lysed with sodium dodecyl sulfate (SDS) sampling buffer (0.05 M Tris–HCl (pH 6.8), 2% SDS, 5.88% 2-mercaptoethanol, 10% glycerol) with 1× protease inhibitor cocktail, 1× phosphatase inhibitor cocktail I, 1× phosphatase inhibitor cocktail II, 1 mM *β*-glycerophosphate and 2.5 mM sodium pyrophosphate. Each sample was electrophoresed in a 6–12% SDS-polyacrylamide gel under reducing conditions and then transferred to a Hybond-ECL nitrocellulose membrane (Amersham Biosciences, Buckinghamshire, UK). The membranes were blocked for 1 h with 20 mM Tris-HCl, pH 7.6, 137 mM NaCl and 0.1% Tween-20 containing 5% skim milk (Snow Brand Milk Products Co., Ltd., Tokyo, Japan) and incubated overnight at 4°C with primary antibody. Immunoreactive protein bands were visualized using an ECL plus detection system with ECL Mini-Camera (Amersham Biosciences).

### 2.6. Statistical Analysis

Results were expressed as means ± SE. Differences were ascertained by an analysis of variance (ANOVA). Multiple comparisons among treatments were checked with Dunnett's test (*P <  .05, **P <  .01).

## 3. Results

### 3.1. Inhibition of Tube Formation, Induction of Apoptosis and Inactivation of ERK1/2 by EEBP

**Figure 1:**
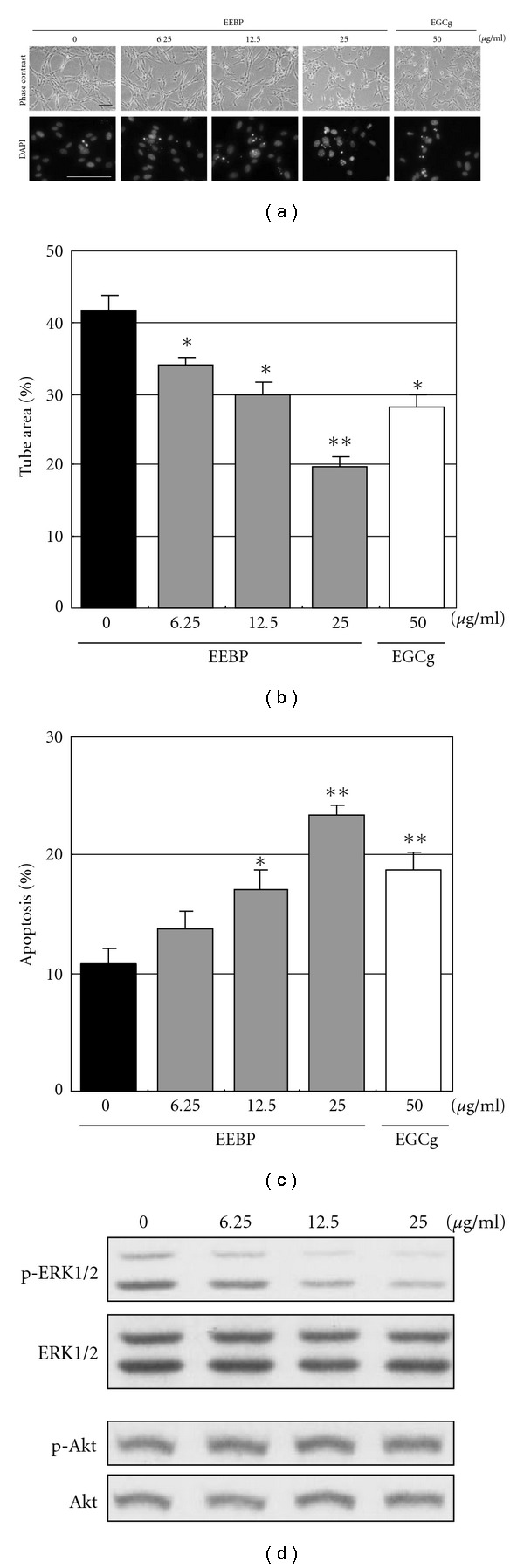
EEBP inhibits tube formation, induces apoptosis and inactivates ERK1/2 in tube-forming HUVECs. (a) HUVECs were sandwiched between two layers of collagen gel and induced to form blood vessel-like tubes. The tube-forming cells were treated with indicated concentrations of EEBP or EGCg for 24 h, fixed and stained with DAPI. Representative photographs are shown. Bar indicates 100 *μ*m. (b) Areas of the formed tubes (area ratios of the tubes per pictured field) and (c) rates of apoptosis (percentage of condensed and fragmented cell nuclei against total cell nuclei) were quantified as described in Materials and methods section. *P <  .05, **P <  .01, as compared to the control. (d) After 12 h treatment with indicated concentrations of EEBP, cellular proteins were collected from tube-forming HUVECs. Changes in phosphorylation state of ERK1/2 at Thr202/Tyr204 and Akt at Ser473 were analyzed by western blotting. Each experiment was repeated at least three times and representative data are shown.

We first investigated whether EEBP had the ability to induce apoptosis in tube-forming endothelial cells compared with EGCg, a well-known tea catechin with an antiangiogenic property. As we previously reported, EEBP had substantial inhibitory effects on angiogenesis *invitro* (Figure 1(a)). Control tube-forming HUVECs, treated with vehicle only, formed a network of capillary-like tubes, which were composed of multiple cells that gathered together and adhered to each other. EEBP reduced the width of tubes and inhibited the elongation of tubes in a concentration-dependent manner. Concurrently, the propolis induced chromatin condensation and nuclear fragmentation, morphological markers of apoptosis, in tube-forming HUVECs in a concentration-dependent manner. The tube areas were 41.8%, 34.0%, 29.9%, 19.9% and 28.0% for control, EEBP (6.25, 12.5 and 25 *μ*g ml^−1^) and EGCg (50 *μ*g ml^−1^ = 109 *μ*M), which were calculated to be 81%, 72%, 48% and 67% for EEBP (6.25, 12.5 and 25 *μ*g ml^−1^) and EGCg (50 *μ*g ml^−1^), respectively, compared to the control (Figure 1(b)). The rates of apoptotic cells were 10.7%, 13.8%, 17.1%, 23.4% and 18.7% for control, EEBP (6.25, 12.5 and 25 *μ*g ml^−1^) and EGCg (50 *μ*g ml^−1^), which were calculated to be a 1.3-, 1.6-, 2.2- and 1.8-fold increase for EEBP (6.25, 12.5 and 25 *μ*g ml^−1^) and EGCg (50 *μ*g ml^−1^), respectively, compared to the control (Figure 1(c)). Thus, it was shown that EEBP at 25 *μ*g ml^−1^ had stronger effects on tube formation inhibition and apoptosis induction than EGCg at 50 *μ*g ml^−1^.

In order to elucidate the molecular mechanisms responsible for apoptosis induction by EEBP, we further analyzed how survival signals, ERK1/2 and Akt, were affected by the propolis. When changes in the activation state of the survival signals were investigated by western blotting, EEBP was shown to inactivate ERK1/2 in a concentration-dependent manner (Figure 1(d)). On the other hand, the propolis had very little effect on Akt activation.

### 3.2. Inhibition of Tube Formation through ERK1/2 Inactivation by U0126

**Figure 2:**
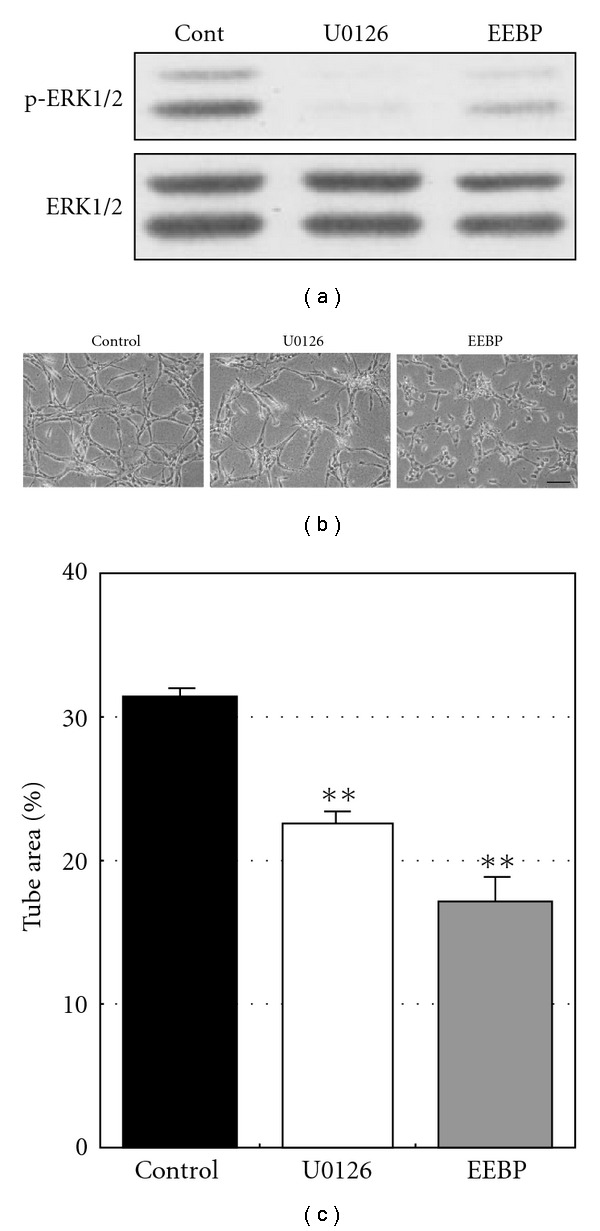
ERK1/2 inactivation by U0126 inhibits tube formation of HUVECs. (a) After 12 h treatment with U0126 (5 *μ*M) or EEBP (25 *μ*g ml^−1^), cellular proteins were collected from tube-forming HUVECs. Changes in phosphorylation state of ERK1/2 at Thr202/Tyr204 were analyzed by western blotting. Each experiment was repeated at least three times and representative data are shown. (b) Tube-forming HUVECs were treated with U0126 (5 *μ*M) or EEBP (25 *μ*g ml^−1^) for 24 h. Representative photographs are shown. Bar indicates 100 *μ*m. (c) Areas of the formed tubes were measured as described in Materials and methods section. Values are expressed as means ± SE of three independent experiments. **P <  .01, as compared to the control.

We next investigated whether ERK1/2 inactivation was actually involved in the regulation of tube formation inhibition by EEBP. We used U0126, a known specific inhibitor of mitogen-activated protein kinase/ERK kinase 1/2 (MEK1/2), to simulate the inhibitory effect of EEBP on ERK1/2. Since MEK1/2 is known to directly phosphorylate ERK1/2, the inhibitor effectively prevents ERK1/2 activation and its downstream signal transduction. U0126 at 5 *μ*m showed a strong inhibitory effect on ERK1/2 activation, which was very close to and slightly stronger than that of EEBP at 25 *μ*g ml^−1^ (Figure 2(a)). The inhibitor also moderately reduced the number of tubes and caused partial fragmentation of the network. EEBP seemed to have an additional inhibitory effect on tube-forming HUVECs, not only reducing the number of tubes and causing partial fragmentation of the network but also inhibiting the elongation of cells (Figure 2(b)). Such observations were further confirmed by quantifying tube area. The areas were 31.3% for control, 22.5% for U0126 and 17.0% for EEBP, which were calculated to be 72% and 54% for U0126 and EEBP, respectively, compared to the control (Figure 2(c)). Thus, it was shown that ERK1/2 inactivation by U0126 was enough to reproduce most of the inhibitory changes induced by EEBP in tube morphology.

### 3.3. Induction of Apoptosis through ERK1/2 Inactivation by U0126

**Figure 3:**
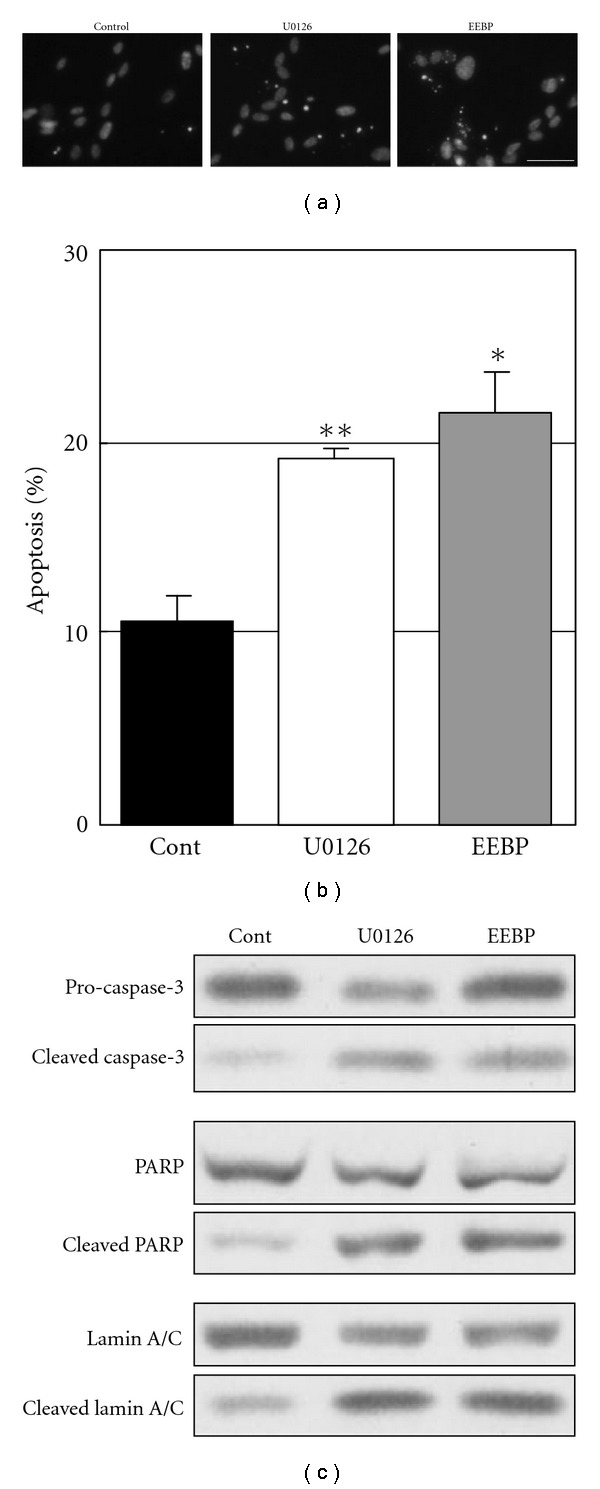
ERK1/2 inactivation by U0126 induces endothelial cell apoptosis at the cellular and molecular levels. (a) Tube-forming HUVECs were treated with U0126 (5 *μ*M) or EEBP (25 *μ*g ml^−1^) for 24 h. The cells were fixed and stained with DAPI. Representative photographs are shown. Bar indicates 50 *μ*m. (b) Rates of apoptosis were quantified. Values are expressed as means ± SE of three independent experiments. *P <  .05, **P <  .01, as compared to the control. (c) Cellular proteins were collected from tube-forming HUVECs that were treated with U0126 (5 *μ*M) or EEBP (25 *μ*g  ml^−1^) for 24 h. Changes in caspase-3, PARP and lamin A/C were analyzed by western blotting. Each experiment was repeated at least three times and representative data are shown.

We further investigated what role ERK1/2 inactivation played in endothelial cell apoptosis induced by EEBP. As shown in Figure 3(a), treatment with either the inhibitor or EEBP resulted in similar increase in the number of apoptotic cells. The rates of apoptotic cells were 10.7% for the control, 19.1% for U0126 and 21.6% for EEBP, which were calculated to be a 1.8- and 2.0-fold increase for U0126 and EEBP, respectively, compared to the control (Figure 3(b)). We then investigated how ERK1/2 inactivation by U0126 or by EEBP affected caspase cascade using western blotting. They both markedly increased the amounts of cleaved forms of caspase-3, PARP and lamin A/C to similar extents (Figure 3(c)). Thus, it was shown that ERK1/2 inactivation by U0126 was enough to reproduce all of the apoptotic changes we observed at both morphological and molecular levels in tube-forming HUVECs induced by EEBP.

## 4. Discussion

**Figure 4:**
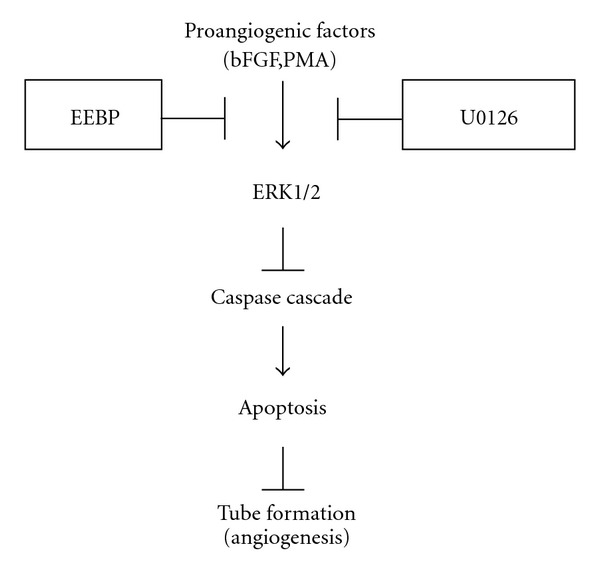
Schematic diagram of angiogenesis suppression by EEBP. Proangiogenic factors such as bFGF and PMA stimulate ERK1/2 signaling. Subsequently, the survival signal inactivates caspase pathway, thereby maintaining cell survival and facilitating angiogenesis. In contrast, EEBP inhibits ERK1/2 activation, thereby activating caspase pathway and inducing apoptosis, which consequently leads to angiogenesis suppression.

We recently reported that propolis inhibited tube formation and induced apoptosis in endothelial cells [12]. In this study, to further elucidate the molecular mechanisms underlying such antiangiogenic effects of propolis, we investigated how EEBP affected two major survival signals, ERK1/2 and Akt. We were able to show that EEBP suppressed ERK1/2 activation, but had very little effect on Akt. We further confirmed that ERK1/2 inactivation was largely responsible for antiangiogenic effects, tube formation inhibition and apoptosis induction, in endothelial cells.  Figure 4 shows proposed antiangiogenic mechanisms by EEBP.

ERK1/2 signaling in endothelial cells has been shown to play an essential role in angiogenesis both *invivo* and *invitro*. It was reported that bFGF and bone morphogenetic protein-4 induced the formation of capillary-like structures by endothelial cells through ERK1/2 activation [25, 26]. In contrast, it was demonstrated that several pharmacological inhibitors, dominant negative constructs and siRNA against Raf/MEK/ERK pathway inhibited angiogenesis *invivo* and tube formation of endothelial cells without affecting Akt activation [27–30]. Furthermore, such inhibitors have also been shown to induce endothelial cell apoptosis *invivo* and *invitro* [31]. Our results are in line with these reports and confirm that ERK1/2 inactivation alone is sufficient to prevent angiogenesis and induce apoptosis in endothelial cells. Thus, we concluded that ERK1/2 inactivation was a major mechanism responsible for antiangiogenic action of EEBP.

Propolis is generally used as an alcohol or water extracts in human applications and not as a single purified compound. In addition, the chemical composition of propolis is known to vary qualitatively and quantitatively depending upon their geographical and botanical origins [23, 32]. Due to such differences, biological activities of propolis also differ depending upon their origins [23, 33–35]. Hence, it is very important to evaluate biological activities of propolis in extracted form with specified geographical and botanical origins and clarified chemical composition [36].

In this study, we showed that EEBP inhibited ERK1/2 activation. We previously reported that Brazilian propolis, collected from *Baccharis dracunculifolia* DC. in Minas Gerais State, were composed mainly of artepillin C, caffeic acid and *p*-coumaric acid [23, 37]. We also reported recently that several constituents of Brazilian and Uruguayan propolis possessed antiangiogenic activities with varying degrees [38]. We would like to further investigate which constituents of the propolis are responsible for ERK1/2 inactivation in endothelial cells. Although EEBP and U0126 had very similar effects on tube formation inhibition and apoptosis induction, it should be noted that the propolis exhibited stronger antiangiogenic activities, such as inhibiting elongation of endothelial cells during tube formation, than those of U0126. Such results suggest a possibility that there might be mechanism(s) other than ERK1/2 inactivation in angiogenesis suppression by EEBP. We would like to further investigate how the propolis affects other signaling pathways involved in angiogenesis and apoptosis. We hope our findings on antiangiogenic effects of propolis will help us improve medical treatment and prevention of human cancer and other angiogenesis-related diseases.

## Funding

Grant-in-aid for Scientific Research from the Ministry of Education, Culture, Sports, Science and Technology of the Japanese Government; grant-in-aid from Japan Society for the Promotion of Sciences (JSPS) [15700470 to T.O.].
